# Decoding Stem Cells: An Overview on Planarian Stem Cell Heterogeneity and Lineage Progression

**DOI:** 10.3390/biom11101532

**Published:** 2021-10-17

**Authors:** M. Dolores Molina, Francesc Cebrià

**Affiliations:** 1 Department of Genetics, Microbiology and Statistics, Faculty of Biology, University of Barcelona, 08028 Barcelona, Spain; 2 Institute of Biomedicine of the University of Barcelona (IBUB), 08028 Barcelona, Spain

**Keywords:** regeneration, planarian, neoblasts, stem cells, progenitors, *piwi*, specification, differentiation, *Schmidtea mediterranea*

## Abstract

Planarians are flatworms capable of whole-body regeneration, able to regrow any missing body part after injury or amputation. The extraordinary regenerative capacity of planarians is based upon the presence in the adult of a large population of somatic pluripotent stem cells. These cells, called neoblasts, offer a unique system to study the process of stem cell specification and differentiation in vivo. In recent years, FACS-based isolation of neoblasts, RNAi functional analyses as well as high-throughput approaches such as single-cell sequencing have allowed a rapid progress in our understanding of many different aspects of neoblast biology. Here, we summarize our current knowledge on the molecular signatures that define planarian neoblasts heterogeneity, which includes a percentage of truly pluripotent stem cells, and guide the commitment of pluripotent neoblasts into lineage-specific progenitor cells, as well as their differentiation into specific planarian cell types.

## 1. Introduction

Some of the main challenges in regenerative medicine are related to the capacity to control stem cell self-renewal and differentiation, as well as to try to induce and/or improve our limited regenerative abilities as mammals. Planarians are one of the few animal models that combine two amazing features: They can regenerate the whole body from a tiny piece of them, and they do it because they maintain a population of adult pluripotent stem cells throughout their lives, referred to as neoblasts [1,2]. Neoblasts are not only needed during regeneration, but they also ensure a continuous cell turnover to replace aged specialized cells and maintain tissue functionality. Therefore, planarians become a good model in which to study in vivo the responses of pluripotent stem cells to a huge variety of external insults such as small injuries, large amputations, presence of toxic substances, long-term starvation and sub-lethal irradiations, among others. Over the last 20 years, significant advances have been made in the planarian field mainly through the use of a large number of different techniques, molecular tools and high-throughput approaches. Consequently, a significant knowledge on how planarians respond to an insult, how regeneration is triggered and how polarity and patterning are achieved during this process has been gathered [3]. Focusing on neoblasts, these stem cells can be easily isolated through FACS [4], can be individually transplanted from one animal to another [5] or even cultured in vitro although with certain current limitations [6]. In addition, neoblasts can be labeled with BrdU and EdU [7,8], which allow for some lineage tracing that can be complemented with cell-type atlases originated from single-cell sequencing experiments [9,10,11,12,13,14,15,16]. From several transcriptomes, dozens of neoblast-specific markers have been identified [12,13,14,16,17,18]. Remarkably, neoblast transcriptomes show several conserved features compared to mammalian stem cells at the level of the specific markers being expressed [19,20].

It is now clear that neoblasts constitute a heterogeneous cell population that includes real pluripotent stem cells as well as a large number of lineage-committed progenitors [21]. By identifying specific markers of all these populations several studies have characterized important genes and pathways required for the differentiation of the distinct mature cell types [12,13,22,23,24,25,26,27,28]. Interestingly, these lineage-committed progenitors appear to maintain a certain plasticity that allows them to switch to a different cell fate depending on the context [29]. Despite all these advances many questions are still under extensive research. For example: What is the role of asymmetric cell division in neoblast self-renewal? Which are the signals that trigger the neoblast response to amputation? How do neoblasts re-adjust to new positional cues? How does metabolic status regulate neoblasts? How and when is a transition between a regenerative and homeostatic context achieved? What is the role of apoptosis in neoblast proliferation and differentiation? Are the gut or the extracellular matrix (ECM) a niche for neoblasts?

In this review, we summarize our current knowledge on some of these questions, as well as on the molecular signatures that define planarian neoblasts’ heterogeneity and guide the commitment of pluripotent neoblasts into lineage-specific progenitors first and mature cells later.

## 2. Planarian Neoblasts Are a Heterogeneous Population of Stem Cells

**Figure 1:**
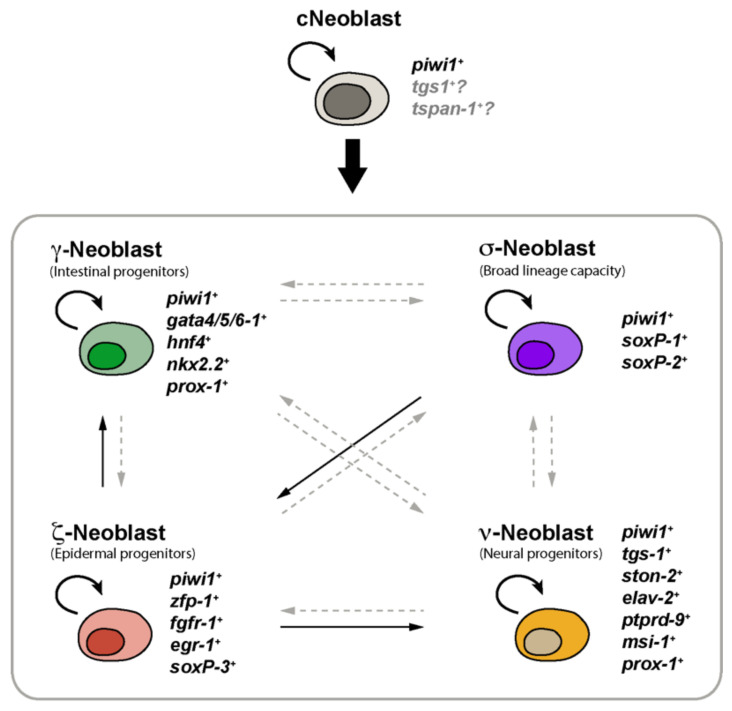
Planarian neoblasts’ heterogeneity. cNeoblasts are pluripotent and can give rise to different subtypes of specialized neoblasts [5] that express a specific subset of transcription factors and will eventually differentiate into distinct planarian tissues such as the gut (γ-neoblasts), epidermis (ζ-neoblasts) or nervous system (ν-neoblasts) [5,12,13]. Some of these specialized neoblasts retain pluripotency and can produce specialized neoblasts for other subtypes [12,29]. Grey discontinued arrows represent untested cell relationships. + symbols indicate expression of the genes. Question marks indicate that the genes cannot be considered exclusive markers of cNeoblasts as are also expressed by other cellular populations.

For many years, planarian stem cells were considered a rather homogeneous cell population. These neoblasts were defined as the only mitotic cells in the asexual strain and were morphologically characterized as small cells that present chromatoid bodies, an elevated nucleus/cytoplasm ratio and that locate in the mesenchymal tissue that fills the space between the planarian organs and the epithelia, among others features [2,30]. Planarian neoblasts are nowadays molecularly defined by the expression of *piwi-1* [31]. In the last decade, however, several studies have revealed that planarian *piwi-1*-expressing cells constitute indeed a heterogeneous population that includes both pluripotent stem cells capable of reconstituting the entire animal [5], as well as specialized progenitors stem cells that co-express tissue-specific transcription factors and contribute to the maintenance and regeneration of specific mature tissues (Figure 1) [21]. Elegant experiments involving single-cell transplantation have demonstrated that a small percentage of planarian neoblasts can be considered truly pluripotent stem cells [5]. These cells, named cNeoblasts (clonogenic neoblasts), are capable of clonal expansion when transplanted individually into planarians that have been irradiated and therefore completely depleted of neoblasts, repopulating the host and differentiating into all of the planarian cell types [5].

Our knowledge regarding neoblasts’ heterogeneity has greatly progressed during the last years [32]. Four main neoblast classes were initially identified and named σ-, γ-, ζ- and ν-neoblasts (Figure 1) [12,13]. σ-neoblasts (*sigma*-neoblasts) were associated with pluripotency, as they proliferate in response to injury, possess a broad lineage capacity and can regenerate other classes of neoblasts, such as the ζ-neoblasts (*zeta*-neoblasts) subtype. These neoblasts were characterized by the elevated expression of genes such as *soxP-1*, *soxP-2*, *soxB-1*, *smad6/7*, *inx-13*, *pbx-1*, *fgfr-4* and *nlk-1* [12]. A second type of stem cells, the ζ-Neoblasts, were considered specialized cells involved in epidermal maintenance and regeneration and express high levels of genes such as *zfp-1*, *fgfr-1*, *p53*, *soxP-3*, *egr-1* and *g6pd* [12]. A third type, the γ-neoblasts (*gamma*-neoblasts), were considered a subclass of the σ-neoblasts that express *gata4/5/6*, *nkx2.2*, *hnf4* and *prox-1* and likely identify the population of intestinal lineage-committed progenitor stem cells [12]. Finally, ν-neoblasts (nu-neoblasts) comprise a neural-committed stem cell population that express low levels of *piwi-1* and neural genes such as *ston-2+* [13]. Several studies have validated the existence of these epidermal, neural and intestinal progenitor stem cells, as well as identified specialized stem cells for other planarian tissues [9,10,16], such as the pharynx [23], eyes [26,27], pigment [28], excretory [22], parenchymal [9,10] or muscle cell types [24,25].

Because of the observation that different levels of *piwi-1* transcript occur in neoblasts and their progeny [13], Zeng and collaborators performed single-cell RNA sequencing and identified at least 12 discrete subpopulations of neoblasts expressing different levels of *piwi-1* [16]. Several of these subpopulations relate to the previously defined subtypes of γ, ζ and ν-neoblasts [12,13]. The previously characterized class of σ-neoblasts associated with pluripotency appeared related to several clusters, suggesting that σ-neoblasts constitute indeed a heterogeneous population. Interestingly, the authors found that a neoblast class marked by the expression of *tgs-1* can be enriched by FACS using an antibody against the cell-surface protein Tetraspanin-1 and suggested that these TSPAN-1^+^ cells are the true pluripotent stem cells that behave as clonogenic neoblasts that self-renew as well as give rise to progenitor cells of the six major cell lineages: epidermal, neural, protonephridia, muscle, pharynx and gut progenitors [16]. It is important to mention, however, that *Tspan-1* does not seem to be exclusively expressed in cNeoblasts, and a notable number of postmitotic cells also express this gene [9,16,33]. In addition, it has been recently proposed that *tgs-1* indeed marks neural-specialized neoblasts as well as postmitotic cells associated with neural fates [29]. Thus, a unique marker of cNeoblasts remains to be characterized, and it has been proposed that these cells may be instead defined by the absence of the expression of any tissue-specific marker [9]. On the other hand, importantly, novel findings suggest that no known neoblast class is uniquely pluripotent, and neoblasts expressing tissue-specific transcription factors from multiple lineages can be clonogenic both after single cell transplantation or under challenging conditions in which stem cells are massively depleted and the few remaining repopulate the entire body [29]. Thus, at least some specialized neoblasts show some degree of plasticity, retain pluripotency and, thereby, should be considered functionally pluripotent and potentially clonogenic (Figure 1) [29]. Therefore, a unique molecular marker of pluripotent neoblasts may not exist, and if it does, it should still be identified.

Although neoblast heterogeneity has been mainly addressed from a lineage potential view, Molinaro and co-workers have recently complemented this analysis and approached the question from a regulatory perspective [33]. In their work, the authors analyzed neoblasts proliferation and identified a population of slow cycling neoblasts that present low transcriptional activity under homeostatic conditions but that enter the cell cycle and mediate regeneration upon injury. The authors propose that these RNA^low^ neoblasts may represent a population of regeneration-reserved neoblasts that relate to both *tspan1^+^* cNeoblasts and other molecularly defined subclasses of neoblasts [33]. In addition to RNA, mitochondrial content has also been associated with neoblast potency [34]. By combining nuclear and mitochondrial staining, Haroon and collaborators conclude that neoblasts with low mitochondrial mass and activity are the true pluripotent stem cells, whereas high mitochondrial mass associates to differentiated cells [34]. Interestingly, it is known that high mitochondrial activity leads to an increased ROS production [35], and ROS production has been shown to be important for the differentiation of stem cells in planarians [36]. In agreement with these observations, experimental inhibition of mitochondrial activity results in increased stemness when assayed by transplantation experiments [34]. Future work will further clarify how RNA and mitochondrial content contribute to neoblast potency and to regeneration.

In summary, advances in single-cell transcriptomics, the isolation of clonogenic TSPAN1^+^ neoblasts, the identification of specific molecular signatures as well as the use of more classical candidate functional approaches have revealed the enormous complexity of the neoblast compartment and its hierarchical organization in these amazing animals (Figure 1).

## 3. From Pluripotent to Lineage-Specialized Stem Cells: Stem Cell Specification

**Figure 2:**
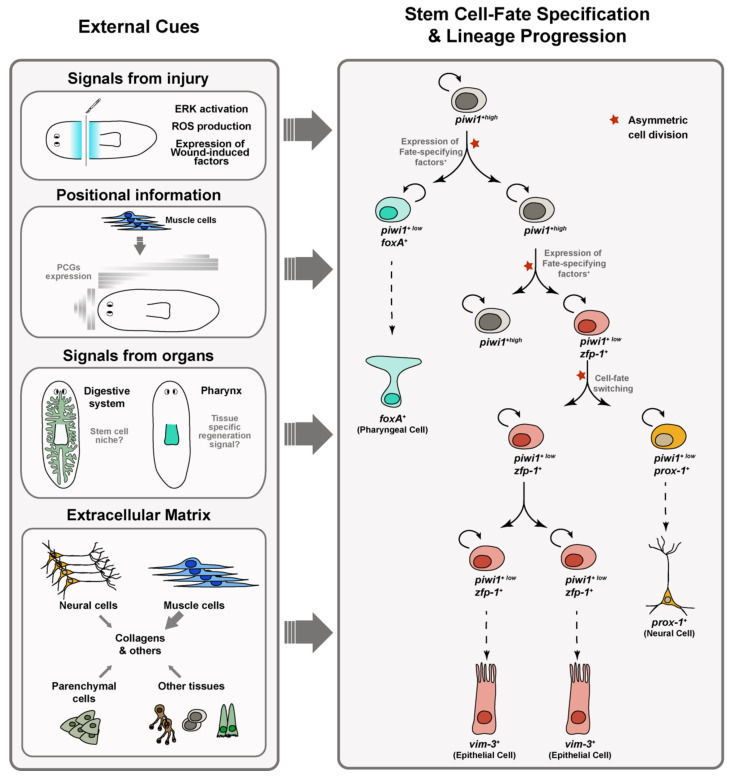
External cues guide planarian stem cell behavior. The balance between cell fate commitment and self-renewal is influenced by external cues such as signals from injury and missing organs and from the extracellular matrix. As differentiation proceeds, cell-fate-specialized progenitors downregulate *piwi-1* expression while the expression of tissue-associated transcription factors increases [9,16,29]. Some specialized neoblasts retain pluripotency and cell fate switching can occur to produce specialized neoblasts for other subtypes [12,29]. Asymmetric cell divisions are marked by a red star.

The expression in neoblasts of tissue-associated transcription factors involved in specifying progenitors has been attributed to neoblast specification during regeneration [21]. As differentiation proceeds, these progenitors downregulate *piwi-1* expression, and the expression of tissue-associated transcription factors increases [9,16,29]. As mentioned in the previous section, the gene expression profiles of multiple stem cell populations have been described in recent years [12,13,14,15,16,21,37,38,39]. In addition, recent single-cell transcriptomic analyses have provided a more complete cell type atlas of the animal and greatly improved our knowledge on the cellular dynamics and putative transition states between stem and differentiated cells in planarians [9,10,11,12,13,14,15,16], allowing the reconstruction of the first lineage tree that connects all planarian cell types to the stem cell compartment [10]. Altogether, these studies have revealed dozens of markers of most planarian cell types. However, the exact cues that maintain the pluripotency of the neoblasts and cause the activation of the genetic programs that specify them into epidermal, gut or neural progenitors, among other cell types, remain poorly understood (Figure 2).

### 3.1. Distinct Responses of Stem Cells to Amputation and Loss of Specific Organs Occur in Planarians

The ability to replace exactly the body regions and tissues that have been damaged or removed by injury is one of the greatest interests in the field of study of planarian regeneration. Several studies have started to investigate how regeneration signals initiate exit from pluripotency and restrict cells to specific differentiation paths to produce the appropriate number and type of specialized neoblasts in response to different regeneration requirements (Figure 2). The rapid activation of ERK after any type of injury has been hypothesized to be one of the earliest requirements to trigger planarian regeneration [40]. ROS production might play a role in this initial ERK activation [41]. Independently of whether injury implies loss of tissue, neoblasts respond by increasing their proliferative rate, and the expression of wound-induced genes is initiated [14,42]. In the case of tissue loss, this initial response is followed by a second proliferative response that will give rise to the regenerative blastema in which the missing tissues will differentiate [43,44]. Cell fate specification during planarian regeneration appears to occur in the stem cells before entering the blastema, suggesting that planarian blastemas are a mosaic of fate-specified progenitors [45]. The intestine might play a niche-like role in modulating neoblast dynamics [7,46,47]. Some neoblast subtypes such as *tgs-1*-expressing neoblasts are located in the parenchyma in close proximity to the gut [16] and knockdown of intestine-enriched transcription factors such as *nkx2.2* or *gata4/5/6-1* or the E3 ligase *wwp1* causes reduced blastema formation and decreased neoblast proliferation [47,48,49]. In addition, the disruption of the integration of new intestinal cells into gut branches by silencing the *egfr-1/ngr-1* ligand receptor pair does alter the number of several progenitor subtypes in addition to increasing the number of gut progenitor cells [50].

Recent studies have started to investigate how stem cells respond to damage or loss of particular organs. The resection of discrete organs such as the eyes or the pharynx combined with the tracking of the specific progenitor cells that target those organs has revealed that the loss of different tissues stimulates distinct behaviors in planarian stem cells. Eye resection, for instance, does not cause eye progenitor amplification, and constant eye progenitor cell production occurs independently of the presence or absence of differentiated eyes [51]. Interestingly, eye progenitor cells are found exclusively in the head region of the planarian, from the eyes to near the pharynx [26,27], suggesting that unidentified extrinsic cues regulate their specification from pluripotent neoblasts distributed uniformly throughout the body. During homeostasis, differentiated eye cells attract these eye progenitor cells for normal cell turnover through an unknown mechanism, while de novo eye formation after resection or amputation depends on the combination of progenitor production with migratory targeting cues and positional information that defines the site of organ regeneration [51,52,53,54]. Similar mechanisms seem to govern de novo pharynx formation and homeostasis [23,53], although in contrast to eye resection, specific removal of the pharynx does trigger a higher proliferation of pharynx progenitors compared to other progenitor types, suggesting that neoblasts sense the identity and the absence of this tissue to launch targeted regeneration [23,55]. Interestingly, these distinct responses of eye and pharynx progenitor pools to the specific loss of the eye and pharynx organs, respectively, have been correlated with the differential activation of ERK signaling. Thus, only pharynx but not eye resection stimulates a proliferative wound response that requires ERK activation and selective progenitor production by stem cell division [55], suggesting that the loss of different organs can activate distinct regenerative mechanisms and stem cell responses in planarians (Figure 2).

### 3.2. The Body-Wall Musculature Serves as a Source of Positional Information in Planarians

The importance of the body-wall musculature for proper regeneration has been established after experiments in which the lack of particular subsets of muscle fibers yield aberrant phenotypes. Thus, *phred-1* is required for proper muscle regeneration which subsequently affects gut patterning [56]. On the other hand, the lack of longitudinal fibers impairs regeneration initiation, whereas the loss of circular fibers generates bifurcated AP axis [25]. In addition, dorsoventral muscles appear to be required for proper medial-lateral patterning [24]. These results point out that different types of body-wall muscle fibers play different roles in wound signaling and patterning to enable regeneration. To our knowledge, no differences between the different fibers of the body-wall musculature depending on their orientation has been described at the structural level. In fact, for example, all of them express the same *myosin heavy chain* gene [57]. However, at the molecular level, different conserved transcription factors are required for the differentiation of distinct sets of body-wall muscle. Although a planarian *myoD* homolog is required for the differentiation of the longitudinal fibers, the differentiation of circular fibers depends on *nkx1-1* [25]. Non-body wall muscle as intestinal and pharynx muscle express a different myosin heavy chain gene [58,59]. Recently, different transcription factors required for the differentiation of non-body wall muscle have also been described. Thus, *foxF-1* is required for the differentiation of both the dorsoventral and the intestinal muscle, whereas the transcription factors *nk4* and *gata4/5/6-2* specify lateral and medial dorsoventral fibers, respectively [24]. Finally, a different *gata4/5/6* homolog is required for the intestinal muscle [24,60].

Planarians constitutively and regionally express dozens of genes that drive instructions for the maintenance and regeneration of the body plan and whose silencing gives rise to defects in polarity and patterning [2]. These genes, known as positional control genes (PCGs), are expressed along the anterior–posterior, dorsal–ventral and medial–lateral axes of the planarian and include members of the Wnt/β-catenin and BMP signaling pathways, as well as transcription factors of the Forkhead and Pbx families, among others [2]. Interestingly, Witchley and co-workers described that PCGs expression occurs in practically all muscle cells from different regions of the planarian, such as the body-wall, gut and pharynx musculature [61,62]. The expression of PCGs in muscle cells is dynamically regulated during regeneration, so muscle cells readjust the expression of these PCGs according to the new position they acquire after amputation [61]. Therefore, the musculature acts not only as a supportive skeletal-like tissue but also as a source of positional information that drives cell fate in planarians (Figure 2) [63]. For example, expression by muscle cells of the tip of the head of *wntA*, *ndk* or of the *fz5/8-4* receptor is important to restrict differentiation of the brain and eyes to the most anterior region of the planarian body [62,64,65,66,67]. Similarly, BMP secreted by dorsal muscle cells regulate the fate of epidermal progenitors by inhibiting the expression of genes associated with ventral epidermal identity [68]. PCGs expressed by muscle cells can also help to guide the regeneration of specific organs, such as the prototypical neural projection pattern of the planarian visual system [69]. In addition to expressing PCGs, muscle cells are also a source of secreted extracellular factors such as the EGF-like ligands EGF-6 and NRG-1 that influence the differentiation of nearby neoblasts into protonephridia and digestive cells, respectively [50,70].

### 3.3. Planarian Muscle Cells Are the Primary Source of Extracellular Matrix

In addition to positional cues, planarian muscle cells are the primary source of extracellular matrix (ECM) and function as connective tissue [71]. The ECM provides the molecular and physical environment for the cell maintenance, self-renewal and differentiation of neoblasts and is required for the proper localization of the cells in the parenchyma (Figure 2) [71,72]. Impaired neoblast migration, proliferation and general tissue growth is observed when the activity of ECM-degrading enzymes is reduced [73,74,75,76]. More recent studies have also revealed the importance of some ECM components such as the EGF repeat-containing genes *megf6* and *hemicentin* to maintain the structure of the basal lamina, restrict the stem cell compartment and retain the parenchymal cell localization of neoblasts and differentiated cells [71,72]. Although some ECM genes are expressed in other tissue types, including intestine, parenchymal cells, neoblasts, neurons and pigment cells, among others [77], muscle cells are the main source of ECM and express core components such as collagen genes [71,77]. Interestingly, these basal membrane collagen and fibrillar collagen genes have been recently shown to play differential roles regulating proliferation during neoblast repopulation, as well as controlling lineage progression. In particular, the basal membrane Type IV collagen *col4-1*, expressed by both neoblasts and muscle cells, is needed for proper planarian tissue maintenance and regeneration, as well as to restrict neoblast number and promote progenitor progression and cell differentiation. These effects are in part mediated by the EGF signaling pathway, suggesting a role of these ECM components in the control of cell fate specification and symmetric versus asymmetric cell division, as mentioned below [77].

### 3.4. Neoblasts Cell Fate Specification Occurs through the Cell Cycle: Asymmetric vs. Symmetric Cell Divisions

A recent study has proposed that planarian cell fate establishment occurs through cell divisions [29]. Neoblasts seem to become specialized around the onset of DNA replication [12,29,78]. The expression of fate-specifying factors correlates with cell-cycle progression, and G1 neoblasts show less expression of the lineage-restricted transcription factors that are associated with cell fate specification and thereby are less specialized than neoblasts in S/G2/M cell-cycles phases or G0 postmitotic cells [29]. In other words, most S/G2/M neoblasts are specialized and express fate-specific transcription factors [29]. This seminal recent study revealed that neoblast specialization is dynamic and not only cell fate establishment but also cell fate switching can occur through cell divisions. Thus, when a specialized neoblast divides, it can give rise to a neoblast that can specialize into the mother fate, into a different fate or even generate an unspecialized daughter cell (Figure 2) [29]. Even though a few pathways of neoblast progressive determination have been documented, especially for the eye and the epidermal lineage [12,26,27,39,54,79,80], these late results challenge the classical view of a categorized stem cell population with specific transcriptional profiles and established differentiation potential and reveal that some of the previously considered lineage-committed neoblasts are instead specialized neoblasts that retain pluripotency [29]. Thus, every subclass of neoblast/progenitor cell is a cloud of likelihood, and instead of a discrete tree-like hierarchy, planarian stem cells can acquire multiple direction lineage biases [81]. As the authors state [29], an important future direction will be to assess the frequency with which fate switching through cell division occurs across multiple specialized neoblasts states.

Neoblast specialization decisions are often not passed on to their neoblast daughters through symmetric, amplifying divisions. Cell fate switching through asymmetric cell divisions frequently occurs in at least one of the daughter cells [29]. During neoblast repopulation, approximately 50% of neoblast divisions are asymmetric and give rise to a neoblast daughter and a post-mitotic daughter cell with high and low expression levels of the neoblast marker *piwi-1*, respectively (Figure 2) [29,82]. Understanding how stem cells control the balance between self-renewal and the production of post-mitotic cells or identifying which signals regulate the asymmetric segregation of cell fate components center some of the current efforts in the study of neoblast biology. Post-transcriptional regulation has been suggested to play an important role in controlling neoblast cell fate decisions and lineage progression [83]. For instance, the influence of alternative splicing for neoblast biology has been evidenced by functional studies of CELF and MBNL RNA binding factors. Planarian neoblasts have a characteristic set of specific mRNA isoforms [84]. Interestingly, Solana and collaborators found that the silencing of *mbln* genes results in the expression of neoblast-specific mRNA isoforms by differentiated cells, while the silencing of *celf* genes causes opposing effects and mRNA isoforms specific of differentiated cells are detected in neoblasts, suggesting a role of alternative splicing on controlling neoblast self-renewal and differentiation [84]. Similarly, based on the increased rate of UsnRNA (Uridylate-rich small nuclear RNA) 3′-processing observed in stem cells compared to differentiated cells, it has been proposed that the cell-type-specific modulation of UsnRNA composition and maturation might contribute to neoblasts’ self-renewal and cell fate choices in planarians [85]. In addition, the RNA-binding translational repressor *mex-3* has been related with stem cell lineage progression and is suggested to act as a repressor of stem cell identity and self-renewal genes in postmitotic progenitors to promote their differentiation. Although the symmetric/asymmetric expression of *piwi-1* transcripts in neoblast doublets has not been analyzed in the context of *mex-3* silencing, MEX3 was proposed as a candidate mediator of asymmetric cell fate in planarians [8]. Interestingly, recent studies have started to identify some of the molecular mechanisms that control the balance of symmetric versus asymmetric cell division of neoblasts. The interaction between the ECM component type IV collagen, the discoidin domain receptor (DDR) and the EGF ligand Neuregulin-7 (NRG-7), via the NRG-7/EGFR pathway, have been found to be important in the process [77]. On the one hand, supporting neurons interact with COL-IV from the extracellular matrix via the DDR1 receptor and regulate the expression of *nrg-7* in neurons [77]. On the other hand, the binding of NRG-7 to its receptor EGFR-3 on neoblasts regulates asymmetric cell divisions and cell fate decision during neoblast repopulation [82]. The EGFR-3 receptor localizes asymmetrically within the cell membrane of neoblasts, which tend to divide symmetrically and show defects in their proliferation and differentiation in *egfr-3* RNAi-silenced planarians [82]. Further investigations would be required to clarify how NRG-7/EGFR-3 signaling regulates neoblast asymmetric division and cell fate choice and whether RNA-binding proteins or mRNA processing play a role in this process.

## 4. Differentiation of Lineage-Committed Stem Cells into Tissue Specific Cell Types

**Figure 3:**
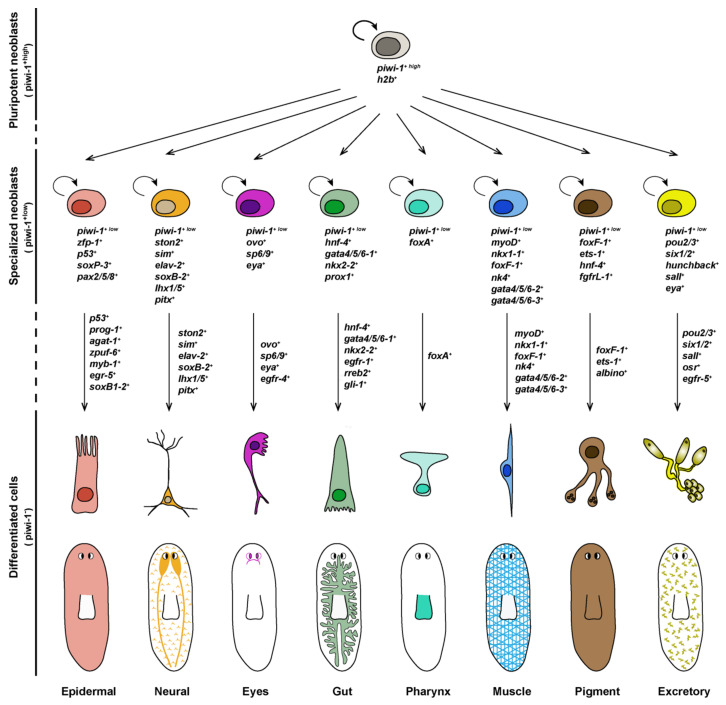
Schematic of planarian stem cell lineages. Markers for each progenitor lineage are indicated. The expression of a subset of these factors is probably further restricted to different cell-specific progenitors within each lineage.

Once individual progenitor populations have been specified, each is characterized by the expression of specific transcription factors often required for their final differentiation into distinct cell fates and organs. For instance, individual progenitors express genes such as *ovo*, *foxA*, *myoD*, *gata4/5/6*, *six-1/2* or *pax6a*, which are also expressed in mature organs such as the eye, pharynx, muscle, gut, excretory system and nervous system, respectively (Figure 3) [21,22,23,25,26,48]. However, researchers have only recently begun to identify the genes and signaling pathways that are activated downstream of these transcription factors during the final stages of differentiation of these progenitors [8,50]. An exhaustive revision of the most important factors required for specification and differentiation of the diverse planarian tissues has been recently reported elsewhere [3,54,86]. Here, we will comment on the latest findings of some key regulators of lineage progression, paying special attention to those important for general differentiation. In addition, a more extensive list of the transcription factors required for differentiation of specific planarian cell types and organs is detailed in Table 1.

The epidermal lineage is among the best characterized in planarians. The epidermis is a monostratified layer of non-ciliated and ciliated epithelial cells that intercalate mucus-secreting cells. The current model of epidermal lineage progression comprises several intermediate cellular stages between epidermal progenitors (the ζ-neoblasts) and mature epidermal cell types: Epidermal progenitors express *zfp-1* and give rise to *prog-1* expressing cells (early epidermal progeny, formerly known as *nb21.11e*) through the activity of *zfp-1*; these *prog-1* cells in turn give rise to *agat-1*-expressing cells (late epidermal progeny), from which cells expressing *zpuf-6* emerge and differentiate into distinct mature epidermal cell types. These different stages of epidermal lineage progression are spatially segregated, with ζ-neoblasts located the deepest in the mesenchyme, and late epidermal progeny cells are closer to the external epithelia [12,39,79]. Epidermal maintenance and regeneration depend on the expression of the transcription factor *zfp-1* by the epidermal progenitors [12], as well as on the activity of *p53*, *sox* and *pax*, which cooperate to regulate genes associated with early epidermal precursor cell differentiation [80]. Zhu and co-workers have recently reported that the specification of the first postmitotic epidermal progenitors depends on *myb-1*, as the silencing of this transcription factor causes a spatiotemporal shift that accelerates epidermal maturation due to the direct specification of stem cell descendants into late epidermal progenitors [88]. The early growth response gene *egr-5*, which co-expresses with *agat-1* in late epidermal progenitors, is required for further progression of the epidermal lineage to mature epidermal cells [79], while the differentiation of subsets of ciliated epidermal cells needs *soxB1-2* (Figure 3) [89].

The planarian digestive system consists of one anterior and two posterior highly ramified gut branches that occupy almost the entire body to ensure that nutrients reach all planarian cells. The intestine is a monostratified epithelium composed of only three cell types: phagocytes, secretory goblet cells and basal cells [9,100], but regionalized gene expression along the mediolateral axis, especially among goblet cells, has been recently described [100]. Gut regeneration is accomplished by the differentiation of stem cells and the remodeling of the pre-existing gastrodermis [104]. Transcriptomic analysis and RNAi screenings have identified a set of genes expressed by intestinal progenitors and their progeny required for processes such as branching, the differentiation of functional phagocytes or neoblast proliferation [5,9,12,16,20,47,100]. Gut progenitor cells have been defined by the expression of the transcription factors *hnf-4* and *gata4/5/6* in *piwi-1*-expressing cells [5,12,21], although the silencing of *gata4/5/6* but not *hnf4* causes defects in gut regeneration and maintenance [48,60]. So far, only the function of the EGFR-1/NRG-1 ligand receptor pair has been shown definitively required for differentiation but not specification of gut progenitor cells into mature phagocytes and goblet cells [50]. Other studies have identified the requirement of the hedgehog signaling effector *gli-1* and the *ras*-*responsive element binding protein 2* (*rreb2*) for the regeneration and maintenance of goblet cells, respectively [100], while the differentiation of functional phagocytes requires the activity of the *ceramide synthase* gene (Figure 3) [47].

Our knowledge on the molecular regulators required for pigment cell lineage development in planarians has progressed rapidly in recent years [105]. The brown body color of freshwater planarians depends on the presence of mature pigment-producing cells that are located in the mesenchyme just underneath the epithelium. The *ets-1* and *foxF-1* transcription factors are found expressed in mature pigment cells [9,28], and its co-expression in some *piwi-1*-positive cells identify pigment stem cell progenitors [9]. Proper pigment lineage progression and dynamics during both homeostasis and regeneration requires the activity of these transcription factors, as well as another Forkhead family member, named *albino*, and the FGF receptor (FGFR)-like molecule *fgfrL-1* [28,101]. *ets-1*, *foxF-1* and *fgfrL-1* play an important role in early pigment progenitor specification, while *albino* may act at later stages of the pigment cell lineage (Figure 3) [28,101,105]. The silencing of any of these factors strongly reduces planarian pigmentation [28]. Although the intermediate stages between pigment progenitor cells and mature pigment-producing cells remain to be characterized, two classes of pigment cell types, dendritic and punctated, have been recently described based on the expression pattern of several pigment markers [28]. Both cell types may represent fully differentiated pigment cell types, but the fact that during regeneration dendritic marker expression precedes punctate marker expression suggests that the former may constitute the progenitor cell type of the latter [28]. Interestingly, the knockdown of *ets-1* during homeostasis primarily affects the punctate cell population, raising the possibility that this factor regulates the shift between dendritic and punctate cell populations [28].

Stem cells should exit the cell cycle to undergo differentiation [106]. The silencing of genes involved in general lineage progression and cell differentiation are predicted to reduce or deplete the number of postmitotic differentiating cells but do not affect the stem cell population. Examples of such factors include the already mentioned MEX3 RNA-binding protein, since the silencing of its expression results in the expansion of the stem cell compartment in parallel with a decrease in the number of lineage-restricted progenitors committed towards eye, brain, pharynx and protonephridia fates [8]. In contrast, the silencing of *apolipoprotein b* triggers stem cell progeny accumulation without affecting neoblast maintenance or proliferation, suggesting that intestine-derived lipids may serve as a source of metabolites needed for neoblast differentiation [107]. The accumulation of key stem cell markers have been shown to occur in neoblasts that fail to differentiate following the silencing of *not1*, a central scaffolding protein of the CCR4-NOT complex involved in mRNA degradation through deadenylation [108]. Similarly, the mRNA base-modificator *m6A* negatively regulates the transcription of histone variants, and its silencing results in the accumulation of undifferentiated cells [109]. PIWI-piRNA complexes are also needed for proper gene expression during neoblast self-renewal and differentiation [110,111,112], and a PIWI homologue inherited from pluripotent neoblasts has been reported to be necessary for transposon silencing and the normal differentiation of descendent cells [113,114]. Other factors such as ROS production [36], the EGF signaling pathway [115], correct lipid metabolism [116] or the activity of genes such as the tumor suppressor *p53* [37,87], the chromatin-remodeling protein *CHD4* [37,117] or the transcription factors *zfp1* and *vasa* [37] also play a general role in planarian cell differentiation, although some of them are also important for the proliferative cell expansion of neoblasts [37]. Finally, several studies have highlighted the importance of epigenetic regulation in the regulation of stem cell differentiation [118,119], as we will further discuss in the following section.

## 5. Post-Translational Modifications and Epigenetic Regulation in Neoblasts

Protein function is often tightly regulated by post-translational modifications (PTMs) that diversify their function through the reversible or irreversible alteration of their structure and properties through biochemical reactions including phosphorylation, glycosylation, ubiquitylation, methylation and acetylation [120]. PTMs, together with other epigenetic modifications, play pivotal roles to regulate stem cell renewal and differentiation [120,121,122]. Those regulatory mechanisms include histone, DNA and chromatin modifications.

Chromatin is important because in addition to compacting DNA it regulates how and when specific molecules such as repair, replication and transcription factors access to the DNA to undertake their function. Thus, gene expression will be mainly regulated through the capacity of these activating and repressing factors to bind their specific DNA targets. Histones undergo many distinct PTMs, including methylation and acetylation [123]. Histone acetylation mainly on lysine side chains promotes the decompaction of chromatin and therefore is associated with the activation of gene expression. Histone acetylation will depend on the balance between two families of enzymes with opposite functions: histone acetyltransferases (HATs) and histone deacetylases (HDACs). For histone methylation the residues involved are lysines and arginines. Whereas lysines can be mono-, di- or trimethylated, arginines can be mono- or dimethylated. Depending on the specific residues that are methylated the effects on gene expression will be opposed. Thus, lysine methylations at H3K4, H3K6 and H3K79 are associated with active genes. whereas those at H3K9, H3K27 and H4K20 are associated with transcriptional repression [123]. It has been shown that in different types of stem cells, developmental genes are marked with both silencing H3K27me3 and active H3K4me3 marks. This bivalent state keeps a poised-transcriptional context that can be rapidly activated upon differentiation [124,125].

In recent years significant progress has been made in order to understand the role of PTMs and epigenetic modifications on planarian regeneration [119,126]. Strand and collaborators have reviewed extensively how post-translational modifications regulate planarian regeneration, mainly phosphorylation, ubiquitination and chromatin modifications [118]. Thus, for example, MAPK (Mitogen-Activated protein kinases) such as JNK and ERK are required for normal regeneration. JNK appears to trigger apoptotic cell-death required for coordinating neoblast proliferation and differentiation, as well as tissue remodeling [127]. On the other hand, ERK has been described as one of the early signals to induce regeneration [40,128] probably through the activation of ROS [41]. Similarly, other kinases involved in neoblast proliferation and differentiation include those from signaling pathways such as PI3K-AKT-TOR [129,130,131], Hippo [132] and the EGFR that has been shown to be required for the differentiation of several cell types [50,115] as well as for asymmetric division [82], as discussed above.

Ubiquitylation and SUMOylation have been shown also to be important for planarian regeneration [118]. Thus, for example, the silencing of the E3 ligase *huwe1* results in an increase in apoptosis and regeneration inhibition despite also inducing an increase in cell proliferation [49]. Similarly, SUMOylation has been reported to regulate cell death and neoblast proliferation probably through the Hedgehog pathway [133].

In addition, several factors involved in chromatin formation and remodeling have been shown to play a role in neoblast function. Thus, a homologue to heterochromatin protein 1 (HP-1) seems to be required to maintain neoblast self-renewal and promote proliferation probably by inducing the expression of *mcm5* [134]. Other important factors in chromatin remodeling belong to the highly conserved SWI/SNF family, which has been also characterized in planarians [135]. The silencing of some components of the two main SWI/SNF complexes BAF and PBAF has revealed their role in neoblast proliferation as well as in the differentiation of the epidermal and muscular lineages [135]. The MLL3/4 histone methyltransferases also participate in stem cell proliferation and the differentiation of the epidermis and neurons [136,137,138]. Moreover, a recent study has found that genes that are mainly silenced in neoblasts but that are activated in their post-mitotic progeny show bivalent H3K4me3 and H3K27me3 marks [139].

**Figure 4:**
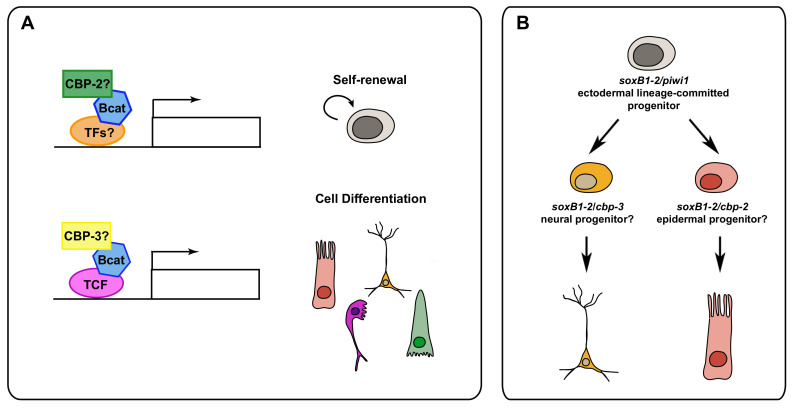
Putative models for the different roles of *cbp-2* and *cbp-3*. Planarian CBP proteins may have diverged functionally to regulate stem cell maintenance and differentiation (**A**) and neuroectodermal lineage progression (**B**).

Recently, two different studies have uncovered the function of the planarian CREB-binding protein (CBP)/p300 family in neoblast biology [126,140]. These transcriptional co-activators play different roles either by acetylating both histone and non-histone proteins (i.e., transcription factors) or serving as protein scaffolds [141,142,143]. On one hand, *cbp-2* appears to be required for stem cell maintenance and planarian survival, as after its silencing, the animals show a significant reduction in the number of neoblasts and proliferative cells and die after a few days of treatment [126,140]. On the other side, *cbp-3* appears to be mainly involved in neoblast differentiation, although some differences are reported in both studies that might be explained by the different RNAi experimental set up as well as the long-term effects of its silencing. Most invertebrates have just one CBP homologue, and a duplication at the base of the vertebrate lineage seems to be the origin of the *p300* gene in that phylum [144]. Remarkably, *S. mediterranea* possess five CBP homologues as a consequence of possible duplication events specific to the phylum Platyhelminthes [126,140]. Even though vertebrates CBP and p300 interact with mainly the same proteins, their functions are not completely redundant [144]. Interestingly, it has been shown that CBP and p300 could regulate embryonic stem cell self-renewal versus differentiation, respectively. These differential roles seem to be mediated by the interaction of CBP and p300 with the Wnt/β-Catenin pathway [145,146]. However, it is still not clear if the function of the β-catenin/CBP complex on stem cell differentiation depends or not on TCF enhancers [147]. Remarkably, *cbp-2* and *cbp-3* appear to play quite complementary functions. Thus, the silencing of *cbp-2* leads to the absence of the first mitotic peak associated with the wound response together with a significant decrease in the number of *piwi-1^+^* cells. In contrast, *cbp-3* RNAi results in a significant increase in the first mitotic peak, as well as of the *piwi-1^+^* population [140]. In addition, for example, *cbp-2(RNAi)* animals show a decrease in the number of gut progenitors, whereas an increase is observed after silencing *cbp-3* [140]. Therefore, it is tempting to speculate that planarian CBP proteins may have diverged functionally in a similar way as vertebrate CBP and p300 appear to regulate stem cell self-renewal and differentiation (Figure 4A).

Another process in which *cbp-2* and *cbp-3* could have complementary functions is that concerning the neuroectodermal lineage. It has been shown recently that a *SoxB1* homologue is expressed in a common neuroectodermal lineage that will give rise to either epidermal or neural cells [89]. Remarkably, a small percentage of *SoxB1*-positive cells co-express either *cbp-2* or *cbp-3* [140], which suggests that planarian CBPs could have a role in specifying the final fates within the neuroectodermal lineage (Figure 4B). In agreement with this putative role of planarian CBPs on neural specification, the inhibition of CBP/p300 in Xenopus leads to an increase in neuronal tissues throughout the embryo at the expense of non-neural tissues [148].

## 6. Conclusions and Future Perspectives

The fine molecular characterization of the planarian neoblasts has uncovered their high level of heterogeneity and a complex hierarchical organization. Through the combination of FACS-based neoblast isolation, single neoblast transplantations and single-cell sequencing, the planarian stem cell compartment has been shown to be constituted by truly pluripotent stem cells that become specialized into multiple lineage progenitors. These distinct cell lineages have been characterized by the expression of specific transcription factors and other genes required for their final differentiation. Importantly, recent studies have started to characterize the onset of neoblast differentiation in relationship to the cell-cycle and their symmetric or asymmetric division. Thus, for example, specialized lineage progenitors show a certain degree of plasticity that allows them to step back towards a pluripotent state and switch cell fate in particularly challenging contexts. Although advances have been made in terms of characterizing the early signals that trigger regeneration (i.e., ERK activation and ROS signaling), how these signals regulate neoblasts behavior remains to be fully understood. Similarly, the muscle fibers provide multiple signals required to regulate the proper patterning during regeneration. How some of these signals act on neoblasts to regulate their proliferation and differentiation is something that requires further studies. In this same line, the possible role of the ECM and the gut as putative niches for neoblasts deserves further investigation. Recent efforts have been made to establish consistent neoblast culture conditions which might be pivotal for the future gene editing of neoblasts as well as to implement transgenesis in these animals [6,149,150]. In addition, some studies have already shown the role that the epigenetic regulation and chromatin remodeling has on neoblasts maintenance and differentiation and will become another important field of study in the near future. Finally, the development of novel tools such as ACME maceration [151] that will allow for a better characterization of planarian cell types and lineages will definitely help to advance in our knowledge on how neoblasts drive regeneration in these amazing animals.

## Figures and Tables

**Table 1:** Transcription factors and signaling molecules required for specification and/or differentiation of planarian cell types.

Lineage	Gene Name	In Situ Expression	*piwi-1^+^* or PIWI-1^+^ Coexpression	RNAi Phenotype	Reference
Epidermal	*zfp-1*	Progenitors	yes	Depletion of epidermal progenitors	[12,37]
	*soxP-3*	Progenitors	n.d.	Reduced early progeny markers	[12,80]
	*egr-1*	Progenitors	yes	n.d.	[12,37]
	*p53*	Progenitors and early progeny	yes	Reduced epidermal progeny	[12,80,87]
	*prog-1*	Early progeny	yes	n.d.	[8,39]
	*myb-1*	Early and late progeny	yes	Absent early progeny fate	[88]
	*agat-1*	Late progeny	no	n.d.	[39]
	*Pax2/5/8*	Late progeny and mature epidermis	n.d.	Reduced early progeny markers	[80]
	*zpuf-6*	Late progeny and mature epidermis	n.d.	n.d.	[79]
	*egr-5*	Late progeny	no	Impaired epidermal differentiation	[79]
	*SoxB1-2*	Neuroectodermal progenitors	yes	Abnormal behavior and movement	[89]
Neural	*ston-2*	Progenitors and different neurons	yes	n.d.	[13]
	*elav-2*	Progenitors and differentiated neurons	yes	n.d.	[13]
	*klf*	Progenitors and differentiated neurons	yes	Absent *cintillo* sensory neurons	[21]
	*pax3/7*	Progenitors and differentiated neurons	yes	Absent *dbh^+^* neurons	[21]
	*neuroD-1*	Progenitors and differentiated neurons	yes	n.d.	[21,90]
	*soxB-2*	Progenitors and differentiated neurons	yes	Reduced neural progenitor expression	[21,91]
	*arrowhead*	Differentiated neurons	n.d.	Defects at the brain commissure	[91]
	*mblk*	Progeny and different neurons	n.d.	Small brain regeneration	[91]
	*tcf/lef-1*	Progenitors and differentiated neurons	yes	Reduced dorsolateral GABA neurons	[21]
	*nkx2.2*	Progenitors and differentiated neurons	yes	Reduced ventromedial neurons	[21]
	*arx*	Differentiated neurons	yes	Reduced ventromedial neurons	[92]
	*Pitx*	Serotonergic and other neurons	yes	Absence of serotonergic neurons	[93,94]
	*lhx1/5*	Serotonergic and other neurons	yes	Absence of serotonergic neurons	[21,93,94]
	*hesl-3*	Progenitors and differentiated neurons	yes	Defects in CNS pattern and organization	[90]
	*sim*	Progenitors and differentiated neurons	yes	Defects in CNS regeneration	[21,90]
	*coe*	Progenitors and differentiated neurons	yes	Defects in CNS size and organization	[90,95]
	*ap-2*	Progenitors and differentiated neurons	yes	Reduced *TrpA*-expressing neurons	[21,42]
	*runt-1*	Neoblasts at wounds	yes	Perturbed *ap-2^+^; sp6-9^+^* expression and neural differentiation	[42]
Eyes	*ovo*	Progenitor and differentiated eye cells	yes	Lack of eye cells	[26]
	*six1/2*	Progenitor and differentiated eye cells	n.d.	Lack of eye cells	[27,42,96,97]
	*eya*	Progenitor and differentiated eye cells	yes	Lack of eye cells	[26,27,42,97]
	*sp6/9*	Progenitor and differentiated PC cells	yes	Lack of PC cells	[27,42]
	*dlx*	Progenitor and differentiated PC cells	yes	Lack of PC cells	[27,42]
	*otxA*	Progenitor and differentiated PH cells	yes	Lack of PH cells	[21,26,27]
	*meis*	Progenitor and differentiated PH cells	yes	Disorganized eye regeneration	[21,26]
	*klf*	Progenitor and differentiated PH cells	yes	Disorganized eye regeneration	[21,26]
	*foxQ2*	Progenitor and differentiated PH cells	yes	Disorganized eye regeneration	[21,26]
	*soxB*	Progenitor and differentiated PH cells	yes	Small eyes and lack anterior PH cells	[21,26]
	*egfr-1*	Differentiated PC cells	n.d.	Reduced number PC cells	[98]
	*egr-4*	Differentiated PH cells	n.d.	Less differentiated and more eye progenitors	[99]
Intestinal	*gata4/5/6-1*	Progenitors and differentiated gut cells	yes	Impaired differentiated gut progenitors	[5,12,48,60]
	*nkx2-2*	Progenitors and differentiated gut cells	n.d.	Impaired gut regeneration and lysis	[12,47]
	*hnf4*	Progenitors and differentiated gut cells	yes	Reduced expression gut markers	[5,12,21]
	*egfr1/nrg-1*	Gut cells	yes	Impaired gut progenitor differentiation	[50]
	*rreb2*	Several gut cell types	n.d.	Impaired goblet cell regeneration	[100]
	*gli-1*	Several gut cell types	n.d.	Impaired goblet cell regeneration	[100]
Pharyngeal	*egfr-1*	Pharynx and others	n.d.	Aberrant pharynx regeneration	[98]
	*FoxA*	Progenit. and differentiated pharynx cells	yes	Impaired pharynx regeneration	[21,23]
Muscular	*nkx1-1*	Progenitors and BWM	yes	Lack of circular fibers and bifurcated AP axis and fused heads	[24,25]
	*myoD*	Progenitors and BWM	yes	Lack of longitudinal fibers and defects in regeneration initiation	[24,25]
	*foxF-1*	Progenitors and non-BWM(DVM, DVL, IM, PM)	yes	Depigmentation, lack of non-BWM and ML defects	[24]
	*nk4*	Progenitors and DVL cells	yes	Reduced DVL markers and ML defects	[24]
	*gata4/5/6-2*	Progenitors and DVM cells	yes	Reduced DVM number and ML defects	[24]
	*gata4/5/6-3*	Progenitors and IM and PM cells	yes	Reduced number of IM cells	[24]
Pigment	*ets-1*	Progenitors and differentiated cells	yes	Depigmentation and reduce markers	[9,28]
	*foxF-1*	Progenitors and differentiated cells	yes	Depigmentation and lack of markers	[9,28]
	*fgfrL-1*	Differentiated pigment cells	n.d.	Depigmentation and reduced punctated marker	[28]
	*albino*	Progenitors and differentiated cells	yes	Depigmentation and lack markers	[28,101]
Excretory	*Six1/2*	Progenitors and tubule cells	yes	Impaired protonephridia regeneration	[22]
	*Pou2/3*	Progenitors and tubule-related cells	yes	Impaired protonephridia regeneration	[22,102]
	*hunchback*	n.d.	n.d.	Impaired protonephridia regeneration	[22]
	*eya*	Progenitors and differentiated cells	yes	Impaired protonephridia regeneration	[22]
	*osr*	Tubule cells	yes	n.d.	[22]
	*SalI*	Progenitors and tubule cells	yes	Edemas and decreased tubule cell number	[22]
	*Egfr-5*	Flame cells	n.d	Absence of flame cells and protonephridia disorganization	[103]

n.d., not determined; CNS, central nervous system; PC, pigment cup cells; PH, photoreceptor cells; BWM, Body wall muscle; DVM, Dorsoventral medial muscle; DVL, Dorsoventral lateral muscle; IM, intestinal muscle; PM, Pharyngeal muscle; AP, anteroposterior; ML, mediolateral.
